# Analysis and verification of the HMGB1 signaling pathway

**DOI:** 10.1186/1471-2105-11-S7-S10

**Published:** 2010-10-15

**Authors:** Haijun Gong, Paolo Zuliani, Anvesh Komuravelli, James R Faeder, Edmund M Clarke

**Affiliations:** 1 Computer Science Department, Carnegie Mellon University, Pittsburgh, PA 15213, USA; 2 Department of Computational Biology, University of Pittsburgh, Pittsburgh, PA 15260, USA

## Abstract

**Background:**

Recent studies have found that overexpression of the High-mobility group box-1 (HMGB1) protein, in conjunction with its receptors for advanced glycation end products (RAGEs) and toll-like receptors (TLRs), is associated with proliferation of various cancer types, including that of the breast and pancreatic.

**Results:**

We have developed a rule-based model of crosstalk between the HMGB1 signaling pathway and other key cancer signaling pathways. The model has been simulated using both ordinary differential equations (ODEs) and discrete stochastic simulation. We have applied an automated verification technique, Statistical Model Checking, to validate interesting temporal properties of our model.

**Conclusions:**

Our simulations show that, if HMGB1 is overexpressed, then the oncoproteins CyclinD/E, which regulate cell proliferation, are overexpressed, while tumor suppressor proteins that regulate cell apoptosis (programmed cell death), such as p53, are repressed. Discrete, stochastic simulations show that p53 and MDM2 oscillations continue even after 10 hours, as observed by experiments. This property is not exhibited by the deterministic ODE simulation, for the chosen parameters. Moreover, the models also predict that mutations of RAS, ARF and P21 in the context of HMGB1 signaling can influence the cancer cell's fate - apoptosis or survival - through the crosstalk of different pathways.

## Background

The cell cycle is strictly regulated and controlled by a complex network of signaling pathways [1], comprised of hundreds of proteins. If some important proteins are mutated or there are defects in the signaling mechanisms, normal cell growth regulation will break down, possibly leading to the occurrence of cancer in the future. Moreover, a number of extracellular proteins can bind to their receptors and activate signaling pathways that promote the proliferation of cancer cells.

The high-mobility group box-1 (HMGB1) protein is a DNA-binding nuclear protein, released actively in response to cytokine stimulation, or passively during cell death [2], and it is present in almost all eukaryotic cells [3-6]. HMGB1 can activate a series of signaling components, including mitogen-activated protein kinases (MAPKs) and AKT, which play an important role in tumor growth and inflammation, through binding to different surface receptors, such as RAGE and TLR2/4. Several studies have shown that elevated expression of HMGB1 occurs in many tumors [7-10] and accelerates cell-cycle progression. Recent *in vitro *studies with pancreatic cancer cells [11] revealed that the targeted knockout or inhibition of HMGB1 and RAGE could increase apoptosis and suppress pancreatic cancer cell growth. This phenomenon has been also observed with lung cancer and other types of cancer cells [8,12].

The HMGB1 signal transduction can influence the cell's fate by two important processes - apoptosis and cell proliferation - which are regulated respectively by the proteins p53 and CyclinE, acting in two different signaling pathways. The protein p53 is one of the most important tumor suppressor proteins: its activation can lead to cell cycle arrest, DNA repair, or apoptosis. Mutations of p53 occur at a frequency of 50% or higher in many different cancer types [13]. CyclinE is a cell cycle regulatory protein which regulates the G1-S phase transition during cell proliferation. Cancer cells often exhibit high expression levels of CyclinE and aberrant CyclinE activity [14]. Many studies have found evidence of crosstalk between the two signaling pathways involving p53 and CyclinE [15]. The crosstalk is regulated by tumor suppressor proteins, including ARF, P21 and FBXW7, which are also frequently mutated in many cancers. In this paper, we ask the following questions: How do these proteins and their mutations change the cell's fate - apoptosis or survival - when HMGB1 signal transduction is activated? Which signaling pathways are fundamental for describing HMGB1 signal transduction, and what mechanisms are responsible to explain recent results linking overexpression of HMGB1 with decrease of apoptosis (and increased cancer cell survival)?

To the best of the authors' knowledge, no computational model has been proposed to investigate the importance of HMGB1 in tumor proliferation. In this work, we construct a simple model of HMGB1 signal transduction to investigate tumorigenesis on the basis of known signaling pathway studies [16-21]. We also constructed a crosstalk network between these known pathways based on hypothetical mechanisms suggested by recent experiments. The HMGB1 pathway is not well understood at the mechanistic level, so our model can provide some insights into the study of HMGB1's roles in tumor proliferation. A series of deterministic and stochastic simulation experiments was conducted to investigate the properties of the HMGB1 pathway.

Finally, we analyze our pathway model against interesting behavorial properties by means of Model Checking techniques. Model Checking is an automated verification technique for hardware and software systems [22]. Recently, there has been growing interest in formal verification of stochastic systems, and, which has recently seen a growing number of applications to biological systems [23-25], by means of Model Checking techniques. The Methods section introduces statistical model checking, which we then apply to validate our pathway model against experimental results from the literature.

## Methods

### HMGB1 signaling pathway

**Figure 1:**
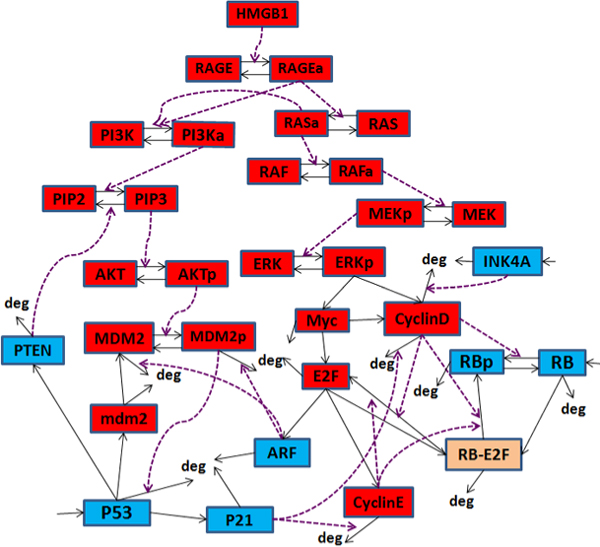
**Schematic view of HMGB1 signal transduction**. Blue nodes represent tumor suppressor proteins; red nodes represent oncoproteins/lipids; brown node represents protein complex formed by oncoprotein E2F and tumor suppressor protein RB. Solid lines with arrows denote protein transcription, degradation or changes of molecular species; dashed lines with arrows denote activation processes.

Our HMGB1 signaling pathway model is illustrated in Fig. 1. It includes 31 molecular species (6 tumor suppressor proteins), 59 chemical reactions, and three different signaling pathways activated by HMGB1: the RAS-ERK, Rb-E2F and p53-MDM2 pathways. Since the interaction between HMGB1 and its receptors TLR and RAGE is not clear at the mechanistic level, RAGE is used to represent all the receptors in our model in order to reduce the number of unknown parameters. We now briefly discuss the three pathways and their crosstalk. We denote activation (or promotion) by →, while inhibition (or repression) is denoted by ⊣.

The *p53-MDM2 pathway *is regulated by a negative feedback loop [26]: PI3K → PIP3 → AKT → MDM2 ⊣ p53 → MDM2, and a positive feedback loop: p53 → PTEN ⊣ PIP3 → AKT → MDM2 ⊣ p53. The protein PI3K is activated by the toll-like receptors (TLR2/4) within several minutes after TLR2/4 activation by HMGB1 [27]. In turn, PI3K phosphorylates the phosphatidylinositol 4,5-bisphosphate (PIP2) to phosphatidylinositol (3,4,5)-trisphosphate (PIP3), leading to phosphorylation of AKT. The unphosphorylated oncoprotein MDM2, which is one of p53's transcription targets [28], resides in the cytoplasm, and cannot enter the nucleus until it is phosphorylated by activated AKT. The phosphorylated MDM2 translocates into the nucleus to bind with p53, inhibiting p53's transcription activity and initializing p53 polyubiquitination [29], which targets it for degradation. Also, p53 can regulate the transcription of PTEN [30], a tumor suppressor protein, which can hydrolyze PIP3 to PIP2, thereby inhibiting the activation of AKT and MDM2.

The *RAS-ERK pathway *is the activation sequence: RAS → RAF → MEK → ERK → CyclinD. Activation of RAGE by HMGB1 leads to RAS activation, which in turn activates its effector protein RAF. Activated RAF will phosphorylate the MEK proteins (mitogen-activated protein kinase kinases (MAPKK)), leading to the phosphorylation of ERK1/2 (also called MAPKs). Activated ERK can phosphorylate some transcription factors which activate the expression of the regulatory protein CyclinD and Myc, enabling progression of the cell cycle through the G1 phase. K-RAS, a member of the RAS protein family, is found to be mutated in over 90% of pancreatic cancers [31].

The *Rb-E2F pathway *is composed of the interactions: CyclinD ⊣ Rb ⊣ E2F → CyclinE ⊣ Rb. The Rb-E2F pathway regulates the G1-S phase transition in the cell cycle during cell proliferation. E2F is a transcription factor that can activate the transcription of many proteins involved in DNA replication and cell-cycle progression [32]. In quiescent cells, E2F is bound by unphosphorylated Rb - a tumor suppressor protein - forming an Rb-E2F complex which inhibits E2F's transcription activity. E2F will be activated and released when its inhibitor Rb is phosphorylated by some oncoproteins (CyclinD and Myc in Fig. 1), leading to the transcription of CyclinE and Cyclin-dependent protein kinase 2 (CDK2) which promote cell-cycle progression. CyclinE, in turn, continues to inhibit the activity of Rb, leading to a positive feedback loop [33-35]. Fig. 1 shows that the activity of CyclinD-CDK4/6 (only CyclinD is shown in Fig. 1) is inhibited by the tumor suppressor protein INK4A, which is inactivated in up to 90% pancreatic cancers [36].

The *crosstalk *between these pathways can influence the cell's fate since the three signaling pathways in HMGB1 signal transduction are not independent. As shown in Fig. 1, the oncoprotein RAS can also activate the PI3K-AKT signaling pathway; the tumor suppressor ARF protein induced by E2F can bind to MDM2 to promote its rapid degradation and stabilize p53. Furthermore, it has been experimentally observed [13] that the p53-dependent tumor suppressor proteins P21 and FBXW7 can inhibit the activity of cyclin dependent kinases (In Fig. 1, we use P21 to represent both P21 and FBXW7's contribution). Mutations of RAS, ARF, P21 and FBXW7 have been found in many cancers [31,36,37]. One of our aims is to investigate how these mutations might influence the cell's fate.

In the HMGB1 model, all substrates are expressed in the number of molecules; proteins with the subscript *"a" *or *"p" *correspond respectively to active or phosphorylated forms of the proteins. For example,

• RAGE (RAGE*_a_*) - inactive (active) form of HMGB1's receptor

• MDM2 (MDM2*_p_*) - unphosphorylated (phosphorylated) MDM2.

We denote the mRNA transcript of MDM2 by *mdm*2. We assume that the total number of active and inactive forms of the RAGE, PI3K, PIP, AKT, RAS, RAF, MEK, and ERK molecules is constant. For example, AKT + AKT*_p _*= AKT*_tot_*, PIP2 + PIP3 = PIP*_tot_*. We sometimes use CD to stand for the CyclinD-CDK4/6 complex, CE for CyclinE, and RE for the Rb-E2F complex.

The p53-MDM2 and RAS-ERK pathways have been studied individually using deterministic ODE methods [16-19,32]. We instead formulated a reaction model corresponding to the reactions illustrated in Fig. 1 in the form of rules specified in the BioNetGen language [38]. We used Hill functions to describe the rate laws governing protein synthesis, including PTEN, MDM2, CyclinD (CD), Myc, E2F and CyclinE (CE). Our choice was motivated by several studies [19,39-41], which showed that transcription rates of these proteins are sigmoidal functions of transcription factor (TF) concentrations with positive cooperative Hill coefficients. We used mass action rules for other types of chemical reactions. Both ODEs and Gillespie's stochastic simulation algorithm (SSA) [42] are used to simulate the model with BioNetGen [38]. Stochastic simulation is important because when the number of molecules involved in the reactions is small, stochasticity and discretization effects become more prominent [43-45]. In the online Additional file 1, we list 23 ordinary differential equations which describe the deterministic HMGB1 model and all the input parameters. The BioNetGen code which implements SSA and ODE models is available at [46].

Since our understanding of many chemical reactions at the mechanistic level is not clear, a large number of parameters involved in these reactions are difficult to estimate based on existing data. We emphasize that in our HMGB1 model the values for some undetermined parameters were chosen in order to produce a qualitative agreement with previous experiments.

### Model Checking

Model Checking [22,47] is one of the leading techniques for the automated verification and analysis of hardware and software systems. Given a high-level behavior specification, a model checker verifies whether a system (or model) satisfies it. A specification might be satisfied by many different models. Thus, model checking is the process of determining whether or not a given system model satisfies (is a model of) a property describing the desired behavior of the system. Mathematically, system models take the form of state-transition diagrams, while some version of temporal logic [48] is used to describe the desired properties (specifications) of system executions. A typical property stated in temporal logic is **G**(*grant_req *→ **F ***ack*), meaning that it is always (**G **= globally) true that a grant request eventually (**F **= future) triggers an acknowledgment. One important aspect of Model Checking is that it can be performed algorithmically - user intervention is limited to providing a system model and a property to check.

The *Probabilistic *Model Checking problem (PMC) is to decide whether a stochastic model satisfies a temporal logic property with a *probability *greater than or equal to a certain threshold. To express temporal properties, we use a logic in which the temporal operators are equipped with *bounds*. For example, the property "CyclinD will always stay below 10 in the next fifty time units " is written as **G**^50^(*CyclinD *< 10). We now ask whether our stochastic system *M *satisfies that formula with a probability greater than or equal to a fixed threshold (say 0.9), and we write M |= *Pr*_≥ 0.9_[**G**^50^(*CyclinD *< 10)]. In the next section, we formally define the temporal logic used in this work, Bounded Linear Temporal Logic [23].

#### Bounded Linear Temporal Logic (BLTL)

Let *SV *be a finite set of real-valued variables, an atomic proposition *AP *be a boolean predicate of the form *e*_1 _~ *e*_2_, where *e*_1 _and *e*_2 _are arithmethic expressions over variables in *SV*, and ~ is either ≥, ≤, <, >, or = . A BLTL property is built over atomic propositions using boolean connectives and bounded temporal operators. The syntax of the logic is the following:

$$\phi ::=AP|\phi_{1}\vee \phi_{2}|\phi_{1}\wedge \phi_{2}|\neg \phi_{1}|\phi_{1}U^{t}\phi_{2}.$$

The bounded until operator *ϕ*_1 _**U***^t ^ϕ*_2_ requires that, *within *time *t*, *ϕ*_2 _will be true and *ϕ*_1 _will hold until then. Bounded versions of the **F **and **G **operators can be easily defined: **F***^t ^ϕ *= *true ***U*^t^****ϕ *requires *ϕ *to hold true within time *t*;G*^t ^ϕ *= ¬**F***^t ^*¬ *ϕ *requires *ϕ *to hold true up to time *t*.

The semantics of BLTL is defined with respect to *traces *(or executions) of a system. In our case, a trace will be the output of a simulation of a BioNetGen stochastic model. Formally, a trace is a sequence of time-stamped state transitions of the form *σ *= (*s*_0_,*t*_0_), (*s*_1_,*t*_1_),..., which means that the system moved to state *s*_*i*+1 _after having sojourned for time *t*_*i *_in state *s*_*i*_. The fact that a trace *σ *satisfies the BLTL property *ϕ *is written as *σ *|= *ϕ. *We denote the trace suffix starting at step *k *by *σ*^*k*^. We have the following semantics of BLTL:

• *σ^k ^*⊨ *AP *if and only if *AP *holds true in state *s_k_*;

• *σ^k ^*⊨ *ϕ*_1 _∧ *ϕ*_2 _if and only if *σ^k ^*⊨ *ϕ*_1 _and *σ^k ^*⊨ *ϕ*_2_;

• *σ^k ^*⊨ *ϕ*_1 _∨ *ϕ*_2 _if and only if *σ^k ^*⊨ *ϕ*_1 _or *σ^k ^*⊨ *ϕ*_2_;

• *σ^k ^*⊨ ¬*ϕ*_1 _if and only if *σ^k ^*⊨ *ϕ*_1 _does not hold;

• *σ^k ^*⊨ *ϕ*_1 _**U^t^***ϕ*_2 _if and only if there exists *i *∈ *N *such that, (a)∑_0 ≤ *l *<*i *_*t*_*k*+l _≤ *t*, (b) *σ*^*k*+*i *^⊨ *ϕ*_2 _and (c) for each 0 ≤ *j *<*i*, *σ*^*k*+*j *^⊨ *ϕ*_1_.

The semantics of BLTL are defined over *infinite *traces, but it can be shown that traces of an appropriate (finite) length are sufficient to decide BLTL properties [49].

#### Statistical Model Checking

We briefly explain *Statistical *Model Checking [50,51], the technique we use for verifying BioNetGen models simulated by Gillespie's algorithm. Statistical Model Checking treats the Probabilistic Model Checking problem as a statistical inference problem, and solves it by randomized sampling of the traces (simulations) from the model. In particular, the PMC problem is naturally phrased as a hypothesis testing problem, *i.e*., deciding between two hypotheses - *M *⊨ *Pr*_≥*θ*_[*ϕ*] versus *M *⊨ *Pr_< θ_*[*ϕ*]. In other words, to determine whether a stochastic system *M *satisfies *ϕ *with a probability *p ≥ θ*, we test the hypothesis *H*_0 _: *p ≥ θ *against *H*_1 _: *p < θ*. Sampled traces are model checked individually to determine whether a given property *ϕ *holds, and the number of satisfying traces is used by a hypothesis testing procedure to decide between *H*_0 _and *H*_1_. Note that Statistical Model Checking cannot guarantee a correct answer to the PMC problem. However, the probability of giving a wrong answer can be made arbitrarily small.

We have introduced a Bayesian sequential hypothesis testing approach and applied it to the verification of rule-based models of signaling pathways and other stochastic systems [23,49]. Sequential sampling means that the number of sampled traces is not fixed a priori, but is instead determined at "run-time ", depending on the evidence gathered by the samples seen so far. This often leads to a significantly smaller number of sampled traces.

Suppose that the stochastic system *M *satisfies the BLTL formula *ϕ *with some (unknown) probability *p*. The key idea behind statistical model checking [50] is that the behavior of *M *(with respect to property *ϕ*) can be modeled by a Bernoulli random variable with success parameter *p*. Such a random variable can be repeatedly evaluated via system simulation in the following way. Let *σ *be a trace of *M*, then the Bernoulli random variable *X *with (conditional) probability mass function:

(1) $$\begin{array}{cc} f(x|p)=p^{x}{\left(1-p\right)}^{1-x} & x\in \{0,1\} \end{array}$$

denotes the outcome of *σ *⊨ *ϕ *(*i.e*., model checking *ϕ *on *σ*). In other words, we have that:

(2) $$X=\{\begin{array}{ll} 1\;\text{with probability}\;p & (\sigma |=\phi ),\\ 0\;\text{with probability}\;1-p & \;(\sigma |=\neg \phi ). \end{array}$$

Therefore, by running a system simulation (*i.e*., a BioNetGen stochastic simulation) and by checking *ϕ *on the resulting trace we can obtain a sample from random variable *X*. When a sample of *X *evaluates to 1 we call it a *success*, otherwise, a *failure*.

Recall that in hypothesis testing we decide between a null hypothesis *H*_0 _and an alternative hypothesis *H*_1_:

(3) H0:p≥θ     H1:p<θ .

The Bayesian approach assumes that *p *is given by a random variable whose distribution is called the *prior distribution*. The prior is usually based on our previous experiences and knowledge about the system.

Since *p *is a probability, we need prior distributions defined over [0,1]. In particular, Beta priors are mathematically convenient to use. They are defined by the following probability density:

(4) $$\begin{array}{cc} \forall u\in [0,1] & g(u,\alpha ,\beta )=\frac{1}{B(\alpha ,\beta )}u^{\alpha -1}{\left(1-u\right)}^{\beta -1} \end{array}$$

where the Beta function *B*(*α, β*) is defined as:

(5) $$B(\alpha ,\beta )=\int_{0}^{1}t^{\alpha -1}{\left(1-t\right)}^{\beta -1}dt.$$

For later use, the Beta distribution function *F*_(*α;β*)_(*u*) of parameters *α, β *is defined as for all *u *∈ [0, 1] as:

(6) $$F_{\left(\alpha ,\beta \right)}(u)=\int_{0}^{u}g(t,\alpha ,\beta )\;dt$$

(7) $$=\frac{1}{B(\alpha ,\beta )}\int_{0}^{u}t^{\alpha -1}{\left(1-t\right)}^{\beta -1}dt.$$

Let *d *= (*x*_1_,..., *x*_*n*_) denote *n *samples of the Bernoulli random variable *X *defined by (2). Let *H*_0 _and *H*_1 _be the hypotheses in (3), and suppose that the *prior probabilities P *(*H*_0_) and *P *(*H*_1_) are strictly positive and satisfy *P *(*H*_0_) + *P *(*H*_1_) = 1. By Bayes's theorem, the *posterior probabilities *of *H*_0 _and *H*_1_, with respect to data *d*, are:

$$\begin{array}{cc} P(H_{i}|d)=\frac{P(d|H_{i})P(H_{i})}{P(d)} & \left(i=0,1\right) \end{array}$$

for every *d *with *P *(*d*) > 0. In our case, *P *(*d*) is always non-zero (there are no impossible *finite *sequences of data). The hypothesis test method is based on the Bayes Factor, that is, the likelihood ratio of the sampled data with respect to the two hypotheses. The Bayes Factor ℬ of sample *d *and hypotheses *H*_0 _and *H*_1 _is

$$ℬ=\frac{P(d|H_{0})}{P(d|H_{1})}$$

and by Bayes' theorem, we have that:

(8) $$ℬ=\frac{P(H_{0}|d)}{P(H_{1}|d)}\;\cdot \;\frac{P(H_{1})}{P(H_{0})}.$$

**Figure 2:**
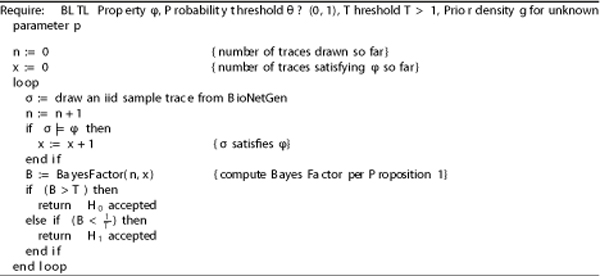
**Statistical Model Checking Algorithm**. The algorithm for Statistical Model Checking is based on Bayesian Hypothesis Testing.

Therefore, *B *can be interpreted as a measure of evidence (given by the data *d*) in favor of *H*_0_. Now, fix a threshold *T *> 1. The algorithm iteratively draws independent and identically distributed (iid) sample traces in the form of BioNetGen stochastic simulations, and checks whether they satisfy *ϕ *(Note that BioNetGen ensures by construction that each simulation, or trace, is actually iid.) After each trace, the algorithm computes the Bayes Factor *B *to check if it has obtained conclusive evidence. The algorithm accepts *H*_0 _if *B *>*T*, and rejects *H*_0 _(accepting *H*_1_) if $ℬ<\frac{1}{T}$. Otherwise $\left(\frac{1}{T}\leq ℬ\leq T\right)$, it continues drawing iid samples. The statistical Model Checking algorithm is shown in Figure 2.

The following Proposition shows that, in our special case of Bernoulli samples, the computation of the Bayes Factor is straightforward.

**Proposition 1**. [49]*The Bayes Factor of H*_0 _: *p *≥ *θ vs. H***_1 _: ***p *<*θ with Bernoulli samples *(*x*_1_,..., *x_n_*) *and Beta prior of parameters α*, *β is:*

$${ℬ}_{n}=\frac{P(H_{1})}{P(H_{0})}\cdot \left(\frac{1}{F_{\left(x+\alpha ,n-x+\beta \right)}(\theta )}-1\right)$$

*where $x=\sum_{i=1}^{n}x_{i}$ is the number of successes in *(*x*_1_,..., *x_n_*) *and F*_(*s,t*)_(·) *is the Beta distribution function of parameters s*, *t*.

The Beta distribution function can be efficiently computed by standard software packages. Thus, no numerical integration is required for the evaluation of the Bayes Factor.

Finally, we must show that the error probability of our decision procedure, *i.e*., the probability that we reject (accept) the null hypothesis although it is true (false), can be bounded.

**Theorem**. [49]*The error probability for the sequential Bayesian hypothesis testing algorithm is bounded above by *$\frac{1}{T}$*where T is the Bayes Factor threshold given as input*.

## Results and discussion

We first conducted a series of deterministic and stochastic simulation experiments to study the properties of our HMGB1 signaling pathway model. Then, we applied the statistical model checking technique to validate some important temporal properties of our HMGB1 model.

### Simulation results

We carried out a baseline simulation for four important proteins - p53, MDM2*_p_*, CyclinD/E - using ODE and stochastic simulation. We set the initial value for the number of HMGB1 molecules to be 10^3^; Table 1 lists all proteins with nonzero initial values; the unlisted proteins are set to 0 initially.

**Table 1:** Initial values for the model

*RAGE*	*PI*3*K*	*PIP*2	*AKT*	*MDM*2	*MDM*2*_p_*	*P*53	*RAS*	*RAF*	*MEK*	*ERK*	*RE*
10^3^	10^5^	10^5^	10^5^	10^4^	2 *× *10^4^	2 *× *10^4^	10^4^	10^4^	10^4^	10^4^	10^5^

Non-zero initial values of the proteins are listed in the table; other unlisted proteins are initially set to 0.

**Figure 3:**
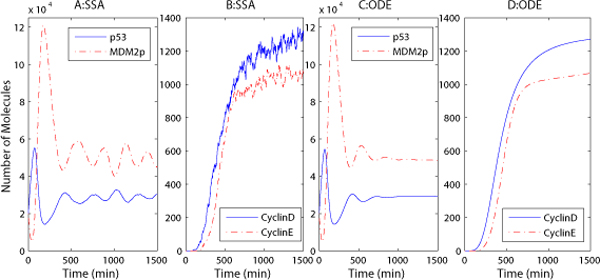
**HMGB1 baseline simulation**. Number of p53, MDM2*_p _*(A, C), CyclinD/E (B, D) molecules versus time for baseline simulations with SSA (A-B) and ODE (C-D) models.

The baseline stochastic simulations in Fig. 3A demonstrate that the expression levels of p53 and MDM2*_p _*oscillate even after 10 hours, when the cell enters the S phase (recall that cells usually remain in phase G1 for about 10 hours before moving to the S phase). However, oscillations are strongly damped in the ODE simulations (Fig. 3C) when the cell proceeds to the S phase, approximately after 10 hours. The stochastic simulation model is thus more consistent with the experimental results of Geva-Zatorsky et al. [52]. In that experiment the authors measured the dynamics of p53 and MDM2*_p _*in human breast cancer cells damaged by γ radiation. It was observed that the oscillations of p53 and MDM2*_p _*expression levels can last more than 72 hours after irradiation.

**Figure 4:**
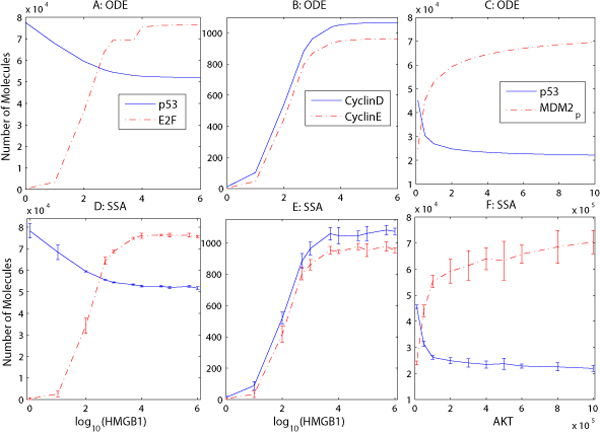
**HMGB1 and AKT sensitivity study**. Overexpression of HMGB1 (A-B, D-E) leads to the increase of oncoprotein E2F and DNA replication proteins CyclinD/E, and to the decrease of p53. Overexpression of AKT (C, F) activated by HMGB1 increases the expression level of MDM2*_p _*and represses p53, using ODE (A-C) and SSA (D-F) simulations.

Fig. 3B and 3D show that the CyclinE protein, which regulates the G1-S phase transition in the cell cycle, reaches its maximum at about 10 hours, after which the cell proceeds with DNA replication (S phase). How does the expression level of HMGB1 and other proteins influence the cell's fate? We varied the levels of HMGB1 and AKT to determine how they affect cell behavior. A number of studies have found that HMGB1 is overexpressed in many cancers, and the overexpression of HMGB1 and its receptors can promote cancer cell proliferation and decrease apoptosis [8,9]. In Fig. 4A-B, we increase the initial values of HMGB1 from 1 to 10^6 ^and measure p53's maximum expression level in phase G1. We then measure the oncoproteins E2F and CyclinD/E's expression levels at 10 hours, which corresponds to the G1-S phase transition point. For the stochastic simulation, the experiment is repeated 10 times per value to compute the mean and standard errors. Fig. 4(A, D) demonstrates that the increase of HMGB1's initial value will lead to a decrease of p53's expression level, but when the number of HMGB1 molecules is over 10^5^, p53 will not continue to decrease. This is because HMGB1 can also activate and increase the expression level of its downstream protein E2F (Fig. 4(A, D)), whose overexpression will activate the transcription of the tumor suppressor protein ARF, which can inhibit MDM2's activity to stabilize p53's level. However, ARF is found to be mutated in up to 80% of pancreatic cancers [36,53]. This means that ARF cannot inhibit the activity of the oncoprotein MDM2, thereby leading to lower levels of the tumor suppressor p53.

Fig. 4(B, E) shows that the cell cycle regulatory proteins CyclinD/E will increase with the elevated expression of HMGB1, a behavior which could be verified by future experiments. Fig. 4(A-B, D-E) explains the experimental discovery that the overexpression of HMGB1 decreases apoptosis and promotes DNA replication and proliferation in cancer cells.

The oncoprotein AKT is overexpressed in many cancers [54]. In Fig. 4(C, F), we first increase the number of unphosphorylated AKT molecules and fix the other proteins' concentration, then measure p53 and MDM2*_p _*'s expression levels at 10 hours in phase G1 after HMGB1 activates its receptor RAGE. Fig. 4(C, F) shows that with the increase of AKT's expression level, p53 is repressed due to the ubiquitination initiated by the overexpressed MDM2*_p_*, which is promoted by the activated and overexpressed AKT protein. The results in Fig. 4(C, F) suggest a way to inhibit tumor cell proliferation and induce tumor cell apoptosis through the inhibition of protein phosporylation events downstream from AKT kinases in the PI3K/AKT pathway, using an AKT kinase inhibitor (such as the drug GSK-690693 [55]).

K-RAS is a member of the RAS protein family. K-RAS mutation and ARF loss occur in more than 80% of pancreatic cancers [36,53]. The P21 and FBXW7 proteins are also frequently mutated in many cancers [37]. ARF and P21 play an important role in the crosstalk between the p53 and Rb pathways. ARF is able to reroute cells with oncogenic damage to p53-dependent fates through binding to MDM2 and targeting its degradation. The p53-dependent tumor suppressor proteins P21 and FBXW7 can inhibit CyclinD/E's activity to prevent the proliferation of cancer cells.

**Figure 5:**
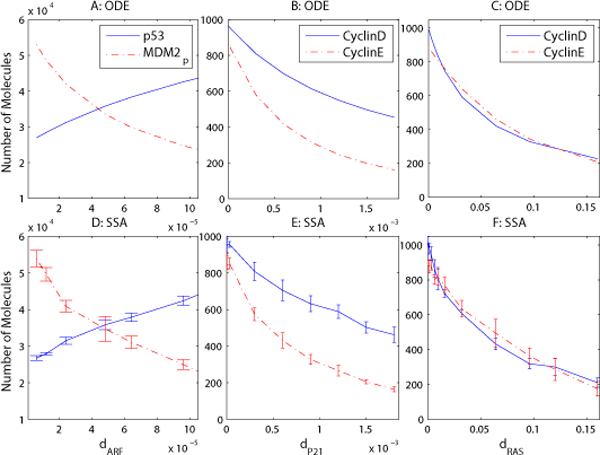
**Mutations of ARF, P21 and RAS affect the cell's fate**. Mutations of the tumor suppressor proteins ARF and P21, and of the oncoprotein RAS affect the cell's fate, using ODE (A-C) and stochastic (D-F) simulations. The mutations of ARF (A, D) and P21 (B, E), and RAS (C, F), which correspond to small *d*_*ARF *_and *d*_*P*21_, and large *d*_*RAS *_values, upregulate the expression level of the oncoproteins MDM2 and CyclinD/E, and downregulate p53's expression level.

Fig. 5 shows how mutations of ARF, P21 and FBXW7, and K-RAS influence tumor suppressor and cell cycle regulatory protein levels at 10 hours in the HMGB1 signaling pathway. We use the MDM2 degradation rate driven by ARF, *d_ARF _*(${d^{\prime }}_{7}$ in the ODE model), to describe ARF mutations. Also, we use the Cyclin degradation rate driven by P21 (*d*_*P*21 _for stochastic simulation, and ${b^{\prime }}_{6}$ for ODE simulation) to describe P21 and FBXW7 mutations. Large *d*_*ARF *_and *d*_*P*21 _values correspond to small mutations of ARF and P21 respectively, while small *d*_*ARF *_and *d*_*P*21 _values correspond to large ARF and P21 mutations in the cell.

Fig. 5(A, D) shows that wild-type ARF (large *d_ARF _*) can decrease the number of MDM2*_p _*molecules and increase p53's expression level to initiate apoptosis even if the cell proceeds to the S phase. Moreover, mutated ARF (smaller *d_ARF _*) can not stabilize p53 expression and prevent the proliferation of cancer cells if HMGB1 is overexpressed. This could explain the phenomenon that ARF loss exists in over 80% of pancreatic cancers [36]. Fig. 5(B, E) demonstrates that CyclinD/E proteins will increase if P21 is mutated (smaller *d*_*P*21_), thereby accelerating cell cycle progression.

K-RAS is mutated in most cancers, especially in pancreatic cancer [31]. The activation of RAS is initiated by HMGB1 and its receptors, and the wild-type RAS can be deactivated by some kinases. Studies have found that the mutated K-RAS can not be deactivated [56], even if HMGB1 is knocked out, so it will continuously activate the downstream signaling pathways which promote cell proliferation. Fig. 5(C, F) shows that with the increase of RAS deactivation rate *d_RAS _*(*b*_1 _in the ODE model), the synthesis of CyclinD/E will be inhibited, but a small deactivation rate of RAS will lead to overexpression of CyclinD/E. The results visualized in Fig. 5 suggest some ways to inhibit cancer cell proliferation through inhibition or deactivation of the signaling pathways involving RAS, Cyclin, and Cyclin-dependent kinases (CDK). Recently, CDK and RAS inhibitor drugs [57-59] have been developed to inhibit tumor growth.

### Verification of the HMGB1 model

We use Statistical Model Checking (SMC) to verify some fundamental properties that our model should satisfy. We test whether the model satisfies a given BLTL property with probability *p *≥ 0.9. We set the threshold *T *= 1000 for the verification, so the probability of a wrong answer is smaller than 10^-3^.

**Property 1: **p53 is normally expressed at low levels in human cells. We verified the following property

$$Pr_{\geq 0.9}[F^{t}(G^{900}(P53<3.3\times 10^{4}))],$$

which informally means that the number of p53 molecules will be less than a threshold value within *t *minutes, and it will always stay below this value during the next 900 minutes. We verified this property with various values of *t *and the results are shown in Table 2.

**Table 2:** Verification of property 1

**Property 1: *Pr***_**≥ 0.9**_**[F**^***t***^**(G**^**900**^**(*P*53 < 3.3 × 10**^**4**^**))]**				
***t*(min)**	**# of Samples**	**# of Successes**	**Result**	**Time (s)**

400	53	49	True	597.59
500	23	22	True	271.76
600	22	22	True	263.79

Property 1 specifies that the number of p53 molecules will be less than a threshold value within *t *minutes, and it will always stay below this value during the next 900 minutes. The initial value of HMGB1 is 10^3^.

**Property 2: **p53's expression level increases quickly in response to various stresses, including the activation of HMGB1. We verified the property

$$Pr_{\geq 0.9}[F^{100}(P53>5.3\times 10^{4})],$$

that is, within 100 minutes p53's level will eventually be larger than 5.3 × 10^4^. SMC accepts this property as true, after sampling 38 traces (of which 37 satisfying traces).

**Property 3: **PI3K will be activated within a few minutes after HMGB1 binds to RAGE. We verified the following property

$$Pr_{\geq 0.9}[F^{20}(PI3K_{a}/PI3K_{tot}>0.5)],$$

which means that half of PI3K will be activated within 20 minutes. We verified this property with various values of HMGB1, and the results are shown in Table 3. If HMGB1 was overexpressed (10^5^), this property was accepted as true (22 satisfying traces). But if the expression level of HMGB1 was very low, the property was rejected.

**Table 3:** Verification of property 3

**Property 3: *Pr *_≥ 0.9_[**F**^20^(*PI*3*K_a_/PI*3*K_tot _>*0.5)]**				
**HMGB1**	**# of Samples**	**# of Successes**	**Result**	**Time (s)**

10^3^	9	0	False	6.49
9 × 10^3^	380	315	False	285.16
10^5^	22	22	True	16.39

Property 3 specifies that half of the PI3K molecules will be activated within 20 minutes when HMGB1 is overexpressed.

**Property 4: **The overexpression of HMGB1 will promote the oncoprotein CyclinE's expression before the G1-S phase transition point, thereby facilitating the G1-S phase transition. We verified the property

$$Pr_{\geq 0.9}\;[\;F^{600}\;(CyclinE\;>900)],$$

that is, the number of CyclinE molecules will eventually exceed 900 within 600 minutes (10 hours). We verified this property with various values of HMGB1 and the results are shown in Table 4.

**Table 4:** Verification of property 4 and 5

**Property 4: *Pr***_≥ 0.9_**[F**^**600**^**(*CyclinE *> 900)]**				**Property 5: *Pr ***_≥ 0.9_**[F**^**600**^**(*CyclinD *> 900)]**			
**HMGB1**	**# of Samples**	**# of Success**	**Result**	***dRAS***	**# of Samples**	**# of Success**	**Result**

10^2^	9	0	False	10^-6^	22	22	True
10^3^	55	16	False	10^-2^	26	5	False
10^6^	22	22	True	10^-1^	9	0	False

Property 4 specifies that the overexpression of HMGB1 will induce the expression of cell regulatory protein CyclinE.

Property 5 specifies that the mutation of RAS will lead to the upregulation of CyclinD.

**Property 5: **Mutation in K-RAS leads to continuous activation of downstream pathways and overexpression of CyclinD in the G1 phase during HMGB1-activated signaling transduction. We verified the property:

$$Pr_{\geq 0.9}\;[\;F^{600}\;(CyclinD\;>900)],$$

with different RAS deactivation rates (*d_RAS_*). The results are presented in Table 4. Properties 4 and 5 show that the overexpression of HMGB1 and mutation of RAS (small *d_RAS _*value) will accelerate the expression of cell regulatory protein CyclinD/E to promote cell proliferation. However, inhibition of HMGB1 and an increase of RAS deactivation rate will prevent tumor growth.

**Property 6: **Within 300 minutes, CyclinE's expression level becomes very low until 50% of RAS has been activated by HMGB1. We verified the property:

$$Pr_{\geq 0.9}[\;(CyclinE\;<10)\;U^{300}(RAS_{a}/RAS_{tot}>0.5)].$$

SMC accepted this property as true (22 satisfying traces).

**Property 7: **HMGB1 could influence the tumor suppressor protein p53's expression level, especially the first peak of p53's concentration in the G1 phase. We verified the following property:

$$Pr_{\geq 0.9}[F^{100}(\frac{p53}{10000}\geq a\wedge F^{100}(\frac{p53}{10000}\leq 0.4))],$$

which informally means that the number of p53 protein molecules in the nucleus will eventually be greater than a threshold value *a *× 10^4 ^within 100 minutes, after which it reduces to a low level within the next 100 minutes. We verified this property with various values of *a *and HMGB1, and the results are shown in Table 5.

**Table 5:** Verification of property 7

**Property 7: *Pr *_≥ 0.9_[**F**^100^(*P *53/10000 ≥ *a *∧ **F**^100^(*P *53/10000 ≤ 0.4))]**					
**HMGB1**	***a***	**# of Samples**	**# of Successes**	**Result**	**Time (s)**

10^3^	5.0	22	22	True	19.74
10^3^	5.5	20	3	False	29.02
10^2^	5.5	22	22	True	19.65
10^2^	6.0	45	12	False	56.27
10	6.5	38	37	True	41.50

Property 7 specifies that the increase of HMGB1's expression level can increase the first peak of p53's concentration in the G1 phase.

## Conclusions

We have presented a reaction network model of the signaling transduction initiated by HMGB1. The model incorporates the contributions from the most important known signaling components of the HMGB1 signal transduction network. The model is expressed in the form of BioNetGen rules, and simulated using ODEs and Gillespie's algorithm under a range of conditions. We used Statistical Model Checking to automatically validate our model with respect to known experimental results.

Our simulations demonstrate a dose-dependent p53 and CyclinE response curve to increasing HMGB1 stimulus. This hypothesis could be tested by future experiments. In particular, overexpression of HMGB1 promotes the cell cycle regulatory proteins E2F and CyclinD/E and inhibits the pro-apoptotic protein p53, leading to increased cancer cell survival and decreased apoptosis. This is consistent with experimental observations in recent studies of cancer cells [11]. We also investigated the roles of different components in the pathway and predicted their activity in response to various conditions. We investigated how mutations of the RAS, ARF and P21 proteins influence the fate of the cancer cell. In particular, parameter variation showed that the mutated RAS increases the expression level of CyclinE, leading to cancer cell proliferation. Mutation or loss of the ARF protein leads to high MDM2 activity and loss of p53 expression in the face of HMGB1 overexpression, resulting in decreased apoptosis. Our model shows that the inhibition (or deactivation) of RAS, Cyclin, and Cyclin-dependent kinases (CDK) might inhibit tumor growth.

Since our proposed model is based on just three signaling pathways, we are far from capturing the entire HMGB1 network dynamics. Studies have found that HMGB1 can not only activate the PI3K-AKT and RAS-ERK pathways, but can also activate the NF*κ*B signaling pathway [27], which regulates many pro-apoptotic and anti-apoptotic proteins' transcription [60]. Since HMGB1 could be released passively during necrosis, there might exist crosstalk between the tumor necrosis factor (TNF) pathway and the HMGB1 pathway. Besides the incorporation of new pathways, recent work has demonstrated that HMGB1 can bind to p53 directly to influence p53-mediated transcriptional activity [61]. A larger network for HMGB1 signal transduction will be explored in our future work.

It has been recently observed that pancreatic tumor cells increase autophagy [11] and release HMGB1 [10] in response to chemotherapy, radiation, and hypoxia, which may promote tumor cell survival. It has been hypothesized that direct inhibition of autophagy may be another way to inhibit tumor growth and enhance the efficacy of cancer therapies [11]. The incorporation of autophagic proteins into the HMGB1 signaling pathway is worth considering in future work.

Although our current model can only qualitatively compare with the experimental behavior, it still provides valuable information about the behavior of HMGB1 signal transduction in response to different stimuli. Future experiments will enable the development of more realistic models. We anticipate that the application of model checking techniques, such as those explored in this work, will facilitate the development of targeted and effective anti-cancer therapies.

## Competing interests

The authors declare that there are no competing interests.

## Authors' contributions

E.M.C., J.R.F., H.G. and P.Z. proposed the project; H.G. and P.Z. wrote the manuscript; H.G. wrote the BioNetGen code and performed the numerical simulations and formal verifications; P.Z. wrote the statistical model checker; A.K. wrote the model checker code for BioNetGen. All authors read and approved the final manuscript.

## Supplementary Material

Additional file 1 **Ordinary differential equations and model parameters**. The PDF file contains all the ordinary differential equations that describe the HMGB1 signal transduction model, the input parameters and their descriptions. Click here for file

## References

[B1] Hanahan D Weinberg RA The hallmarks of cancer Cell 2000 100 57 70 10.1016/S0092-8674(00)81683-9 10647931

[B2] Scaffidi P Misteli T Bianchi M Release of chromatin protein HMGB1 by necrotic cells triggers inflammation Nature 2002 418 191 195 10.1038/nature00858 12110890

[B3] Bonaldi T Talamo F Scaffidi P Ferrera D Porto A Bachi A Rubartelli A Monocytic cells hyperacetylate chromatin protein HMGB1 to redirect it towards secretion EMBO J 2003 22 5551 5560 10.1093/emboj/cdg516 14532127 PMC213771

[B4] Wang H Bloom O Zhang M Vishnubhakat J Ombrellino M Frazier A Yang H HMG-1 as a late mediator of endotoxin lethality in mice Science 1999 285 248 10.1126/science.285.5425.248 10398600

[B5] Dumitriu IE Baruah P Valentinis B Release of high mobility group box 1 by dendritic cells controls T cell activation via the receptor for advanced glycation end products The Journal of Immunology 2005 174 7506 7515 15944249 10.4049/jimmunol.174.12.7506

[B6] Semino C Angelini G Poggi A Rubartelli A NK/iDC interaction results in IL-18 secretion by DCs at the synaptic cleft followed by NK cell activation and release of the DC maturation factor HMGB1 Blood 2005 106 609 616 10.1182/blood-2004-10-3906 15802534

[B7] Sparvero L Asafu-Adje D Kang R Tang D Amin N RAGE (Receptor for Advanced Glycation Endproducts), RAGE ligands, and their role in cancer and inflammation Journal of Translational Medicine 2009 7 17 10.1186/1479-5876-7-17 19292913 PMC2666642

[B8] Ellerman JE Brown CK de Vera M Zeh HJ Billiar T Masquerader: high mobility group box-1 and cancer Clinical Cancer Research 2007 13 2836 2848 10.1158/1078-0432.CCR-06-1953 17504981

[B9] Lotze MT Tracey K High-mobility group box 1 protein (HMGB1): nuclear weapon in the immune arsenal Nature Reviews Immunology 2005 5 331 342 10.1038/nri1594 15803152

[B10] Vakkila J Lotze MT Inflammation and necrosis promote tumour growth Nature Reviews Immunology 2004 4 641 648 10.1038/nri1415 15286730

[B11] Kang R Tang D Schapiro NE Livesey KM Farkas A Loughran P Lotze MT Zeh HJ The receptor for advanced glycation end products (RAGE) sustains autophagy and limits apoptosis, promoting pancreatic tumor cell survival Cell Death Differ 2010 17 4 666 676 10.1038/cdd.2009.149 19834494 PMC3417122

[B12] Brezniceanu ML Volp K Bosser S Solbach C Lichter P HMGB1 inhibits cell death in yeast and mammalian cells and is abundantly expressed in human breast carcinoma FASEB Journal 2003 17 1295 1297 12759333 10.1096/fj.02-0621fje

[B13] Vogelstein B Lane D Levine AJ Surfing the p53 network Nature 2000 408 307 310 10.1038/35042675 11099028

[B14] Minella AC Grim JE Welcker M Clurman BE p53 and SCFFbw7 cooperatively restrain cyclin E-associated genome instability Oncogene 2007 26 6948 6953 10.1038/sj.onc.1210518 17486057

[B15] Pestov DG Strezoska A Lau LF Evidence of p53-dependent cross-talk between ribosome biogenesis and the cell cycle: effects of nucleolar protein Bop1 on G1/S transition Molecular and Cellular Biology 2001 21 13 4246 4255 10.1128/MCB.21.13.4246-4255.2001 11390653 PMC87085

[B16] Wee KB Aguda BD Akt versus p53 in a network of oncogenes and tumor suppressor genes regulating cell survival and death Biophysical Journal 2006 91 857 865 10.1529/biophysj.105.077693 16648169 PMC1563780

[B17] Wee KB Surana U Aguda BD Oscillations of the p53-Akt Network: Implications on Cell Survival and Death PloS One 2009 4 e4407 10.1371/journal.pone.0004407 19197384 PMC2634840

[B18] Ciliberto A Novak B Tyson J Steady States and Oscillations in the p53/Mdm2 Network Cell Cycle 2005 4 3 488 493 15725723 10.4161/cc.4.3.1548

[B19] Zhang T Brazhnik P Tyson J Exploring mechanisms of the DNA-damage response: p53 pulses and their possible relevance to apoptosis Cell Cycle 2007 6 85 e10 10.4161/cc.6.16.4577 17245126

[B20] Puszynski K Hat B Lipniacki T Oscillations and bistability in the stochastic model of p53 regulation Journal of Theoretical Biology 2008 254 452 465 10.1016/j.jtbi.2008.05.039 18577387

[B21] Bottani S Grammaticos B Analysis of a minimal model for p53 oscillations Journal of Theoretical Biology 2007 249 235 245 10.1016/j.jtbi.2007.04.026 17850824

[B22] Clarke EM Grumberg O Peled DA Model Checking 1999 MIT Press

[B23] Jha SK Clarke EM Langmead CJ Legay A Platzer A Zuliani P A bayesian approach to model checking biological system CMSB, Volume 5688 of LNCS 2009 218 234

[B24] Langmead CJ Generalized Queries and Bayesian Statistical Model Checking in Dynamic Bayesian Networks: Application to Personalized Medicine CSB 2009 201 212

[B25] Rizk A Batt G Fages F Soliman S On a continuous degree of satisfaction of temporal logic formulae with applications to systems biology CMSB, Volume 5307 of LNCS 2008 251 268

[B26] Larris S Levine AJ The p53 pathway: positive and negative feedback loops Oncogene 2005 24 2899 2908 10.1038/sj.onc.1208615 15838523

[B27] van Beijnum JR Buurman WA Griffioen AW Convergence and amplification of toll-like receptor (TLR) and receptor for advanced glycation end products (RAGE) signaling pathways via high mobility group B1 Angiogenesis 2008 11 91 99 10.1007/s10456-008-9093-5 18264787

[B28] Barak Y Juven T Haffiner R Oren M mdm2 expression is induced by wild type p53 activity EMBO J 1993 12 461 468 8440237 10.1002/j.1460-2075.1993.tb05678.x PMC413229

[B29] Haupt Y Maya R Kasaz A Oren M Mdm2 promotes the rapid degradation of p53 Nature 1997 387 296 299 10.1038/387296a0 9153395

[B30] Blanco-Aparicio C Renner O Leal J Carnero A PTEN, more than the AKT pathway Carcinogenesis 2007 28 1379 1386 10.1093/carcin/bgm052 17341655

[B31] Downward J Targeting RAS signalling pathways in cancer therapy Nature Reviews Cancer 2003 3 11 22 10.1038/nrc969 12509763

[B32] Yao G Lee TJ Mori S Nevins J You L A bistable Rb-E2F switch underlies the restriction point Nature Cell Biology 2008 10 476 482 10.1038/ncb1711 18364697

[B33] Sherr CJ McCormick F The RB and p53 pathways in cancer Cancer Cell 2002 2 103 112 10.1016/S1535-6108(02)00102-2 12204530

[B34] Sears R Nevins J Signaling networks that link cell proliferation and cell fate The Journal of Biological Chemistry 2002 277 11617 11620 10.1074/jbc.R100063200 11805123

[B35] Nevins JR The Rb/E2F pathway and cancer Human Molecular Genetics 2001 10 699 703 10.1093/hmg/10.7.699 11257102

[B36] Bardeesy N DePinho RA Pancreatic cancer biology and genetics Nature Reviews Cancer 2002 2 12 897 909 10.1038/nrc949 12459728

[B37] Mao J Perez-losada J Wu D DelRosario R Tsunematsu R Nakayama KI Brown K Bryson S Balmain A Fbxw7/Cdc4 is a p53-dependent, haploinsufficient tumour suppressor gene Nature 2004 432 775 779 10.1038/nature03155 15592418

[B38] Hlavacek WS Faeder JR Blinov ML Posner RG Hucka M Fontana W Rules for modeling signal-transduction system Sci STKE 2006 2006 344 re6 **re6** 10.1126/stke.3442006re6 16849649

[B39] Bolouri H Computational Modeling of Gene Regulatory Networks: a primer 2008 Imperial College Press

[B40] Yang H Hsu C Hwang M An Analytical Rate Expression for the Kinetics of Gene Transcription Mediated by Dimeric Transcription Factors Journal of Biochemistry 2007 142 135 144 10.1093/jb/mvm151 17652330

[B41] Alon U An introduction to systems biology: design principles of biological circuits 2007 Chapman & Hall

[B42] Gillespie DT A general method for numerically simulating the stochastic time evolution of coupled chemical reactions Journal of Computational Physics 1976 22 4 403 434 10.1016/0021-9991(76)90041-3

[B43] Gong H Sengupta H Linstedt A Schwartz R Simulated De Novo Assembly of Golgi Compartments by Selective Cargo Capture during Vesicle Budding and Targeted Vesicle Fusion Biophysical Journal 2008 95 1674 1688 10.1529/biophysj.107.127498 18469086 PMC2483775

[B44] Gong H Guo Y Linstedt A Schwartz R Discrete, continuous, and stochastic models of protein sorting in the Golgi apparatus Phys Rev E Stat Nonlin Soft Matter Phys 2010 81 011914 20365406 10.1103/PhysRevE.81.011914 PMC5367640

[B45] Lipniacki T Hat T Faeder JR Hlavacek WS Stochastic effects and bistability in T cell receptor signaling Journal of Theoretical Biology 2008 254 110 122 10.1016/j.jtbi.2008.05.001 18556025 PMC2577002

[B46] HMGB1 BioNetGen Code http://www.cs.cmu.edu/~haijung/research/HMGB1model.bngl

[B47] Clarke EM Emerson EA Sifakis J Model checking: algorithmic verification and debugging Commun ACM 2009 52 11 74 84 10.1145/1592761.1592781

[B48] Pnueli A The Temporal Logic of Programs FOCS 1977 46 57

[B49] Zuliani P Platzer A Clarke EM Bayesian statistical model checking with application to Simulink/Stateflow verification HSCC 2010 243 252 full_text

[B50] Younes HLS Simmons RG Probabilistic Verification of Discrete Event Systems Using Acceptance Sampling CAV, Volume 2404 of LNCS 2002 223 235

[B51] Younes HLS Simmons RG Statistical probabilistic model checking with a focus on time-bounded properties Information and Computation 2006 204 9 1368 1409 10.1016/j.ic.2006.05.002

[B52] Geva-Zatorsky N Rosenfeld N Itzkovitz S Milo R Sigal A Dekel E Yarnitzky T Liron Y Polak P Lahav G Alon U Oscillations and variability in the p53 system Molecular Systems Biology 2006 2 2006.0033 10.1038/msb4100068 16773083 PMC1681500

[B53] Hahn WC Weinberg RA Modelling the molecular circuitry of cance Nature Reviews Cancer 2002 2 331 341 10.1038/nrc795 12044009

[B54] Altomare D Wang H Skele K Rienzo AD Klein-Szanto A Godwin A Testa J AKT and mTOR phosphorylation is frequently detected in ovarian cancer and can be targeted to disrupt ovarian tumor cell growth Oncogene 2004 23 5853 7 10.1038/sj.onc.1207721 15208673

[B55] Rhodes N Heerding D Duckett D Characterization of an Akt kinase inhibitor with potent pharmacodynamic and antitumor activity Cancer Research 2008 68 2366 10.1158/0008-5472.CAN-07-5783 18381444

[B56] van Krieken J KRAS mutation testing for predicting response to anti-EGFR therapy for colorectal carcinoma: proposal for an European quality assurance program Virchows Arch 2008 453 417 431 10.1007/s00428-008-0665-y 18802721

[B57] McInnes C Progress in the evaluation of CDK inhibitors as anti-tumor agents Drug Discovery Today 2008 13 1920 875 881 10.1016/j.drudis.2008.06.012 18639646

[B58] Malumbres M Pevarello P Barbacid M Bischoff J CDK inhibitors in cancer therapy: what is next? Trends in Pharmacological Sciences 2008 29 16 10.1016/j.tips.2007.10.012 18054800

[B59] Rotblat B Ehrlich M Haklai R Kloog Y The Ras inhibitor farnesylthiosalicylic acid (Salirasib) disrupts the spatiotemporal localization of active Ras: a potential treatment for cancer Methods in Enzymology 2008 439 467 489 full_text 18374183 10.1016/S0076-6879(07)00432-6

[B60] Lipniacki T Paszek P Brasier A Luxon B Kimmel M Crosstalk between p53 and nuclear factor-kB systems: pro-and anti-apoptotic functions of NF-kB Journal of Theoretical Biology 2004 228 195 215 10.1016/j.jtbi.2004.01.001 15094015

[B61] Imamura T Izumi H Nagatani G Ise T Nomoto M Iwamoto Y Kohno K Interaction with p53 enhances binding of cisplatin-modified DNA by high mobility group 1 protein J Biol Chem 2001 276 7534 7540 10.1074/jbc.M008143200 11106654

