# Supplementary material for: Analysis and verification of the HMGB1 signaling pathway
Source: BMC Bioinformatics. 2010 Oct 15;11(Suppl 7):S10. doi: 10.1186/1471-2105-11-S7-S10 (PMC2957678; doi:10.1186/1471-2105-11-S7-S10)
Supplement: Additional file 1 — Ordinary differential equations and model parameters. The PDF file contains all the ordinary differential equations that describe the HMGB1 signal transduction model, the input parameters and their descriptions. [file 1471-2105-11-S7-S10-S1.pdf]

Additional file 1 for

Analysis and verification of the HMGB1 signaling pathway

Haijun Gong*[1], Paolo Zuliani*[1] , Anvesh Komuravelli[1] , James R. Faeder[2], Edmund M. Clarke[1]

Email: Haijun Gong*- haijung@cs.cmu.edu; Paolo Zuliani*- pzuliani@cs.cmu.edu; Anvesh Komuravelli - anvesh@cs.cmu.edu; James R. Faeder - faeder@pitt.edu; Edmund M. Clarke - emc@cs.cmu.edu;

*Corresponding author

Ordinary differential equations and model parameters

Ordinary differential equations

The HMGB1 model is described by 23 ordinary differential equations.

$$
\begin{aligned}
\frac{d}{dt}RAGE_a(t) &= k_1 RAGE(t)HMGB1 - d_1 RAGE_a(t)\\
\frac{d}{dt}PI3K_a(t) &= (k_2 RAGE_a(t) + k_2' RAS_a(t))PI3K(t) - d_2 PI3K_a(t)\\
\frac{d}{dt}PIP3(t) &= k_3 PI3K_a(t)PIP2(t) - d_3 PTEN(t)PIP3(t)\\
\frac{d}{dt}AKT_p(t) &= k_4 PIP3(t)AKT(t) - d_4 AKT_p(t)\\
\frac{d}{dt}PTEN(t) &= \frac{k_5 P53(t)^3}{K_1^3 + P53(t)^3} - d_5 PTEN(t)\\
\frac{d}{dt}mdm2(t) &= \frac{k_6 P53(t)^3}{K_1^3 + P53(t)^3} - d_6 mdm2(t)\\
\frac{d}{dt}MDM2(t) &= k_7 mdm2(t) + d_8 MDM2_p(t) - (d_7 + k_8 AKT_p(t) + d_7' ARF(t))MDM2(t)\\
\frac{d}{dt}MDM2_p(t) &= k_8 AKT_p(t)MDM2(t) - d_8 MDM_p(t) - (d_8' + d_7' ARF(t))MDM2_p(t)\\
\frac{d}{dt}P53(t) &= k_9 - d_9 P53(t) - d_9' MDM2_p(t)P53(t)\\
\frac{d}{dt}P21(t) &= \frac{k_{10} P53(t)^2}{K_1^2 + P53(t)^2} - d_{10} P21(t)\\
\frac{d}{dt}RAS_a(t) &= a_1 RAGE_a(t)RAS(t) - b_1 RAS_a(t)\\
\frac{d}{dt}RAF_a(t) &= a_2 RAS_a(t)RAF(t) - b_2 RAF_a(t)\\
\frac{d}{dt}MEK_p(t) &= a_3 RAF_a(t)MEK(t) - b_3 MEK_p(t)
\end{aligned}
$$

$$
\begin{aligned}
\frac{d}{dt}ERK_p(t) &= a_4 MEK_p(t)ERK(t) - b_4 ERKp(t) \\
\frac{d}{dt}Myc(t) &= \frac{a_5 ERK_p(t)}{K_2 + ERK_p(t)} - b_5 Myc(t) \\
\frac{d}{dt}CD(t) &= \frac{a_6 ERK_p(t)}{K_2 + ERK_p(t)} + \frac{a_6' Myc(t)}{K_2 + Myc(t)} - (b_6 + b_6' P21(t) + b_6'' INK4A(t))CD(t) \\
\frac{d}{dt}RE(t) &= a_7 RB(t)E2F(t) - b_7 RE(t) - b_7'(CD(t) + CE(t))RE(t) \\
\frac{d}{dt}RB_p(t) &= (a_8 RB(t) + b_7' RE(t))CD(t) - (b_8 + b_8')RB_p(t) + b_7' CE(t)RE(t) \\
\frac{d}{dt}RB(t) &= a_9 + b_8 RB_p(t) - a_8 CD(t)RB(t) - (a_7 E2F(t) + b_9)RB(t) \\
\frac{d}{dt}E2F(t) &= \frac{a_{10} Myc(t)}{K_2 + Myc(t)} + b_7' CD(t)RE(t) - (a_7 RB(t) + b_{10})E2F(t) + b_7' CE(t)RE(t) \\
\frac{d}{dt}ARF(t) &= \frac{a_{11} E2F(t)}{K_3 + E2F(t)} - b_{11} ARF(t) \\
\frac{d}{dt}CE(t) &= \frac{a_{12} E2F(t)}{K_3 + E2F(t)} - b_{12} CE(t) - b_6' CE(t)P21(t) \\
\frac{d}{dt}INK4A(t) &= a_{13} - b_{13} INK4A(t)
\end{aligned}
$$

HMGB1 model parameters

We list all the parameters' values used in the HMGB1 model and some references. Some parameters' values were either abstracted and converted from the literature, or estimated from some experimental results. Since the understanding of many chemical reactions at the mechanistic level is not clear, for example, the interaction between HMGB1 and RAGE, a large number of parameters could not be estimated from the current available data. So, we chose some parameters' values which produce qualitative agreement with existing experimental results.

	Value	Reaction Rate description & Refs		Value	Reaction rate description & Refs
k_1	2×10^{-5}	HMGB1 activates RAGE	d_1	2×10^{-2}	RAGE inactivation
k_2	5.4×10^{-5}	RAGE activates PI3K	d_2	5×10^{-3}	PI3K inactivation rate [1]
k_3	3×10^{-6}	PI3K phosphorylates PIP2	d_3	3.5×10^{-5}	PTEN dephosphorylates PIP3 [2]
k_4	1.2×10^{-7}	PIP3 phosphorylates AKT [3]	d_4	1.2×10^{-2}	AKT_p dephosphorylation [4,5]
k_5	28.0	PTEN production [2,6]	d_5	6×10^{-3}	PTEN degradation [2,7]
k_6	32	mdm2 transcription [2]	d_6	1.8×10^{-2}	mdm2 degradation [2]
k_7	30	mdm2 translation [2]	d_7	1.4×10^{-2}	MDM2 degradation [8–10]
k_8	4×10^{-7}	$MDM2_p$ phosphorylation [2,10]	d_8	3×10^{-2}	$MDM2_p$ dephosphorylation [2,5]
k_9	1.2×10^{3}	p53 production [11]	d_9	1.2×10^{-2}	p53 degradation [9,11]
k_{10}	8.0	p21 production	d_{10}	1.8×10^{-2}	p21 degradation [12]
a_1	2×10^{-5}	RAGE activates RAS	b_1	1.6×10^{-3}	RAS inactivation [1]
a_2	10^{-7}	RAS activates RAF [1,13]	b_2	9×10^{-3}	RAF inactivation [13]
a_3	4×10^{-7}	RAF activates MEK	b_3	1.8×10^{-3}	MEK inactivation [1]
a_4	8×10^{-7}	MEK activates ERK	b_4	2×10^{-3}	ERK inactivation
a_5	90	Myc production	b_5	1.2×10^{-2}	Myc degradation [14,15]
a_6	50	CyclinD synthesis driven by ERK	b_6	3.5×10^{-2}	CyclinD degradation [16,17]
a_7	10^{-6}	RB binds to E2F [18]	b_7	5×10^{-4}	RB-E2F degradation [18]
a_8	10^{-5}	RB phosphorylation	b_8	6×10^{-6}	RB_p dephosphorylation
a_9	40	RB production	b_9	5×10^{-4}	RB degradation [19]
a_{10}	60	E2F production	b_{10}	4×10^{-4}	E2F degradation [20]
a_{11}	30	ARF production	b_{11}	5×10^{-3}	ARF degradation [21,22]
a_{12}	170	CyclinE production	b_{12}	3.5×10^{-2}	CyclinE degradation [23,24]
a_{13}	40	INK4A production	b_{13}	3×10^{-3}	INK4A degradation [25]
k'_2	3×10^{-7}	RAS activates PI3K [13]	d'_8	1.3×10^{-2}	$MDM2_p$ degradation [2,10]
a'_6	90	CyclinD synthesis driven by Myc	b'_7	6×10^{-6}	Cyclin D/E dissociate RB-E2F
b'_8	4×10^{-4}	RB_p degradation [19]	d'_9	6×10^{-7}	MDM2$_p$ drives p53 degradation [2,8]
d'_7	6.5×10^{-6}	ARF inhibits MDM2 [22]	b''_6	3×10^{-6}	INK4A inhibits Cyclin D
b'_6	3×10^{-7}	P21 inhibits Cyclin D/E	K_1	8×10^{4}	Michaelis-Menten constant [2]
K_2	2×10^{3}	Michaelis-Menten constant	K_3	3×10^{5}	Michaelis-Menten constant

References

1. Brown K, Hill C, Calero G, Myers C, Lee K, Sethna J, Cerione R: **The statistical mechanics of complex signaling networks: nerve growth factor signaling**. *Physical Biology* 2004, **1**:184–195.
2. Puszynski K, Hat B, Lipniacki T: **Oscillations and bistability in the stochastic model of p53 regulation**. *Journal of Theoretical Biology* 2008, **254**:452–465.
3. Giri L, Mutalik V, Venkatesh K: **A steady state analysis indicates that negative feedback regulation of PTP 1 B by Akt elicits bistability in insulin-stimulated GLUT 4 translocation**. *Theoretical Biology and Medical Modelling* 2004, **1**:2.
4. Qiu D, Mao L, Kikuchi S, Tomita M: **Sustained MAPK activation is dependent on continual NGF receptor regeneration**. *Development, Growth & Differentiation* 2004, **46**:393.
5. Kholodenko B: **Negative feedback and ultrasensitivity can bring about oscillations in the mitogen-activated protein kinase cascades**. *European Journal of Biochemistry* 2000, **267**:1583.
6. Stambolic V, Macpherson D, Sas D, Lin Y, Snow B, Jang Y, Benchimol S, Mak T: **Regulation of PTEN transcription by p53**. *Molecular Cell* 2001, **8**:317.
7. Georgescu M, Kirsch K, Akagi T, Shishido T, Hanafusa H: **The tumor-suppressor activity of PTEN is regulated by its carboxyl-terminal region**. *Proceedings of the National Academy of Sciences* 1999, **96**:10182.
8. Wee KB, Aguda BD: **Akt versus p53 in a network of oncogenes and tumor suppressor genes regulating cell survival and death**. *Biophysical Journal* 2006, **91**:857–865.

9. Bar-Or R, Maya R, Segel L, Alon U, Levine A, Oren M: **A plausible model for the digital response of p53 to DNA damage**. *Proceedings of the National Academy of Sciences* 2000, **97**:11250.

10. Ciliberto A, Novak B, Tyson J: **Steady States and Oscillations in the p53/Mdm2 Network**. *Cell Cycle* 2005, **4**(3):488–493.

11. Ma L, Wagner J, Rice J, Hu W, Levine J, Stolovitzky G: **A plausible model for the digital response of p53 to DNA damage**. *Proceedings of the National Academy of Sciences* 2005, **102**:14266.

12. Roy S, Kaur M, Agarwal C, Tecklenburg M, Sclafani R, Agarwal R: **p21 and p27 induction by silibinin is essential for its cell cycle arrest effect in prostate carcinoma cells**. *Molecular Cancer Therapeutics* 2007, **6**:2696.

13. Orton R, Adriaens M, Gormand A, Sturm O, Kolch W, Gilbert D: **Computational modelling of cancerous mutations in the EGFR/ERK signalling pathway**. *BMC Systems Biology* 2009, **3**:100.

14. Sears R, Nuckolls F, Haura E, Taya Y, Tamai K, Nevins J: **Multiple Ras-dependent phosphorylation pathways regulate Myc protein stability**. *Genes and Development* 2000, **14**:2501.

15. Yeh E, Cunningham M, Arnold H, Chasse D, Monteith T, Ivaldi G, Hahn W, Stukenberg T, Shenolikar S, Uchida T, Counter C, Nevins J, Means A, Sears R: **A signalling pathway controlling c-Myc degradation that impacts oncogenic transformation of human cells**. *Nature Cell Biology* 2004, **6**:308.

16. Diehl J, Zindy F, Sherr C: **Inhibition of cyclin D1 phosphorylation on threonine-286 prevents its rapid degradation via the ubiquitin-proteasome pathway**. *Gene and Development* 1997, **11**:957.

17. Sherr C, Roberts J: **CDK inhibitors: positive and negative regulators of G1-phase progression**. *Genes and Development* 1999, **13**:1501.

18. Yao G, Lee TJ, Mori S, Nevins J, You L: **A bistable Rb-E2F switch underlies the restriction point**. *Nature Cell Biology* 2008, **10**:476–482.

19. Mihara K, Cao X, Yen A, Chandler S, Driscoll B, Murphree A, T'Ang A, Fung Y: **Cell cycle-dependent regulation of phosphorylation of the human retinoblastoma gene product**. *Science* 1989, **246**:1300.

20. Helin K: **Regulation of cell proliferation by the E2F transcription factors**. *Current Opinion in Genetics and Development* 1998, **8**:28.

21. Kuo M, den Besten W, Bertwistle D, Roussel M, Sherr C: **N-terminal polyubiquitination and degradation of the Arf tumor suppressor**. *Genes & Development* 2004, **18**:1862.

22. Proctor C, Gray D: **Explaining oscillations and variability in the p53-Mdm 2 system**. *BMC Systems Biology* 2008, **2**:75.

23. Clurman B, Sheaff R, Thress K, Groudine M, Roberts J: **Cyclin E-CDK2 is a regulator of p27Kip1**. *Genes and Development* 1996, **10**:1979.

24. Won K, Reed S: **Activation of cyclin E/CDK2 is coupled to site-specific autophosphorylation and ubiquitin-dependent degradation of cyclin E**. *EMBO Journal* 1996, **15**:4182.

25. Ha T, Segev D, Barbie D, Masiakos P, Tran T, Dombkowski D, Glander M, Clarke T, Lorenzo H, Donahoe P, Maheswaran S: **Mulerian inhibiting substance inhibits ovarian cell growth through an Rb-independent mechanism**. *The Journal of Biological Chemistry* 2000, **275**:37101.
